# Distribution Model Reveals Rapid Decline in Habitat Extent for Endangered Hispid Hare: Implications for Wildlife Management and Conservation Planning in Future Climate Change Scenarios

**DOI:** 10.3390/biology13030198

**Published:** 2024-03-20

**Authors:** Imon Abedin, Tanoy Mukherjee, Ah Ran Kim, Hyun-Woo Kim, Hye-Eun Kang, Shantanu Kundu

**Affiliations:** 1 Agricultural and Ecological Research Unit, Indian Statistical Institute, Kolkata 700108, India; 2 Elephant Research & Conservation Division, Aaranyak, Guwahati 781028, India; 3 Research Center for Marine Integrated Bionics Technology, Pukyong National University, Busan 48513, Republic of Korea; 4 Department of Marine Biology, Pukyong National University, Busan 48513, Republic of Korea; 5 Institute of Marine Life Science, Pukyong National University, Busan 48513, Republic of Korea; 6 Institute of Fisheries Science, Pukyong National University, Busan 48513, Republic of Korea

**Keywords:** lagomorphs, species distribution modelling, protected area planning, transboundary, climate change

## Abstract

**Simple Summary:**

The hispid hare, *Caprolagus hispidus*, an endangered small mammal species, occupies a range from the southern lowlands of Nepal, India, and Bhutan. Despite its endangered status, there is limited knowledge about its distribution throughout its range and protected areas. This study aimed to identify factors influencing its distribution and determine suitable protected areas both in present and future climatic scenarios, considering bioclimatic, habitat, anthropogenic, and topographic factors. The findings are crucial for shaping effective long-term conservation strategies. The assessment of remaining habitats within protected areas provides critical insights into suitable habitat and climate refugia for this species.

**Abstract:**

The hispid hare, *Caprolagus hispidus*, belonging to the family Leporidae is a small grassland mammal found in the southern foothills of the Himalayas, in India, Nepal, and Bhutan. Despite having an endangered status according to the IUCN Red List, it lacks studies on its distribution and is threatened by habitat loss and land cover changes. Thus, the present study attempted to assess the habitat suitability using the species distribution model approach for the first time and projected its future in response to climate change, habitat, and urbanization factors. The results revealed that out of the total geographical extent of 188,316 km^2^, only 11,374 km^2^ (6.03%) were identified as suitable habitat for this species. The results also revealed that habitat significantly declined across its range (>60%) under certain climate change scenarios. Moreover, in the present climate scenario protected areas such as Shuklaphanta National Park (0.837) in Nepal exhibited the highest mean extent of habitat whereas, in India, Dibru-Saikhowa National Park (0.631) is found to be the most suitable habitat. Notably, two protected areas in Uttarakhand, India, specifically Corbett National Park (0.530) and Sonanandi Wildlife Sanctuary (0.423), have also demonstrated suitable habitats for *C. hispidus*. Given that protected areas showing a future rise in habitat suitability might also be regarded as potential sites for species translocation, this study underscores the importance of implementing proactive conservation strategies to mitigate the adverse impacts of climate change on this species. It is essential to prioritize habitat restoration, focused protection measures, and further species-level ecological exploration to address these challenges effectively. Furthermore, fostering transboundary collaboration and coordinated conservation actions between nations is crucial to safeguarding the long-term survival of the species throughout its distribution range.

## 1. Introduction

Lagomorphs, comprising pikas, hares, and rabbits, are pivotal members of ecosystems globally, contributing to ecological dynamics and serving diverse roles [1]. They constitute a significant mammalian order of scientific importance, such as serving as a primary carnivore food source and essential component within food webs [2]. Lagomorphs exhibit wide distribution across various habitats, including tropical forests, temperate regions, steppes, plateaus, deserts, and even Arctic areas spanning Eurasia, Africa, North America, and Central America, encompassing all continents except Antarctica [2,3,4]. Inhabiting elevations ranging from sea level to over 5000 m and spanning from the equator to 80°N latitude, these organisms thrive in various environmental conditions [3]. The taxonomy of Lagomorpha has recently undergone revisions, resulting in the classification of all species into two families: Ochotonidae and Leporidae. Ochotonidae comprises a single genus, *Ochotona*, encompassing 25 species of small, social pikas predominantly found in high-latitude and high-altitude regions. Conversely, Leporidae encompasses 32 species of large, solitary hares and jackrabbits within the genus *Lepus*, along with 30 species of medium-sized, semi-social, fossorial rabbits distributed across 10 genera. A substantial portion of lagomorphs is documented in the International Union for Conservation of Nature (IUCN) Red List of Threatened Species, with a noteworthy proportion being highly range-restricted species. Fourteen species in particular are categorized under IUCN Criteria B, with an estimated extent of occurrence of less than 20,000 km^2^ [2,3]. The hispid hare, *Caprolagus hispidus*, is a highly elusive mammal belonging to the Leporidae family and is classified as ‘Endangered’ according to the IUCN Red List of Threatened Species. It is also listed under Appendix-I of the Convention on International Trade in Endangered Species of Wild Flora and Fauna (CITES) [5] and holds Scheduled-I status under the Indian Wildlife (Protection) Act, 1972 [6,7]. Additionally, it is one of the lagomorph species protected by the US Endangered Species Act [8]. Notably, this species holds the distinction of being the world’s rarest mammal within a monotypic genus [5,9]. Recognizable by its coarse dark-brown fur on the dorsal side, featuring a mix of black and brown hair, and a ventral coat that is brown on the chest and whitish on the abdomen, *C. hispidus* is commonly referred to as the ‘bristly rabbit’ [10].

The species inhabited a historical range stretching from the southern foothills of the Himalayas in Uttar Pradesh (India), across Nepal, and into West Bengal to Assam (India), with its southern extent reaching Dhaka in Bangladesh. However, its present distribution is constrained to the isolated tropical grasslands found in Nepal, India, and Bhutan [11]. There have been scarce published records of captures or confirmed sightings of *C. hispidus* since its initial type specimen was described by Blyth in 1845, leading some authorities to fear the species had become extinct until it was jointly rediscovered with the sympatric Pygmy Hog (*Sus salvanius*) in northern Assam in 1971 [7,12,13]. However, the confirmation of its continued existence came in 1971 with the live capture of a specimen from the Barnadi Wildlife Sanctuary (BaWLS) in Assam. Presently, the hispid hare displays a fragmented distribution in southern Asia, including Nepal, Bhutan, Bangladesh, and India [11,14], within an elevation range of 100–250 m [5].

The hispid hare predominantly inhabits tall grasslands marked by early-succession vegetation, often situated alongside riverbanks [15]. Its primary habitats are the floodplain grasslands of the Terai region, distinct from other dry and scrub grasslands commonly observed across the subcontinent [5,10,16]. These floodplains or alluvial grasslands boast tall grass species such as *Saccharum spontaneum*, *Imperata cylindrical*, *Desmostachya bipinnata*, *Narenga porphyrocoma*, and *Themeda arundinacea* [10,11,17,18]. The hispid hare predominantly consumes thatch shoots and roots, biting them off at the base and stripping off the outer sheaths before ingestion [19]. These grasslands represent dynamic ecosystems that offer essential resources to sustain various flora and fauna, serving as habitats for numerous endangered species [14,20]. However, these grasslands face threats primarily from natural succession, overgrazing by cattle, unregulated thatch collection, and uncontrolled burning [21,22]. Consequently, these grasslands provide increasingly limited refuge areas for small mammals, including the hispid hare [5,23].

Due to the limited ecological studies and inventories focusing on small mammals in the grasslands of the Indian subcontinent, particularly in comparison with large mammals [24], there remains a significant gap in understanding the population status and ecology of the hispid hare throughout its distribution range [9,17,25]. Consequently, vital information concerning its distribution and the factors influencing its habitat utilization is missing [11]. The species faces a continual decline across its range due to escalating anthropogenic activities such as urbanization and human settlement development. However, the lack of comprehensive data regarding its distribution and habitat preferences impedes targeted conservation efforts [5]. The species is negatively affected by habitat destruction resulting from agriculture, deforestation, urbanization, flood control measures, and irrigation practices, compounded by the detrimental effects of seasonal burning, overgrazing, and the depletion of remaining preferred habitats [14,15]. Despite its rediscovery over three decades ago, the hispid hare continues to receive minimal attention from researchers and conservationists in the region. This insufficiency of baseline information poses challenges to conservation endeavors and assessments of its conservation status [14].

Due to the absence of previous studies of the species distribution across its entire range, formulating comprehensive conclusions regarding its responses to various climatic and anthropogenic factors proves challenging. Enhancing our comprehension of the synergistic impacts of climate change and land cover will offer deeper insights into the varying levels of susceptibility exhibited by different species to climate change. The alterations in climate are anticipated to exert significant influences on species ecology and distribution, leading to pronounced effects on terrestrial biodiversity [2,26,27,28]. While climate naturally undergoes changes, recent land cover changes have accelerated this process, raising concerns [2]. Moreover, acquiring knowledge about both the present and anticipated future conditions of habitats is imperative for effective conservation and management planning. Future climate change is expected to exert substantial effects on species’ niches, representing the biotic and abiotic conditions necessary for species persistence. Species are projected to respond by adapting their bioclimatic niche, migrating to suitable habitats to maintain their existing niche, or experiencing range limitations and subsequent declines in population, potentially leading to local or global extinctions under future scenarios [29,30]. The multitude of factors contributing to habitat loss may encompass diverse external influences prevalent in previous periods [31].

Managing the species at both habitat and landscape levels relies on establishing the species’ range and identifying suitable habitats [32]. Species distribution models (SDMs) are important tools for assessing the likelihood of species occurrence within specific geographic areas, providing essential information for habitat management and conservation initiatives [33,34,35,36]. In recent years, the integration of ecophysiological models has been crucial in SDM projections for various vertebrate species, aiding in understanding range shifts in response to climate change [37,38,39,40]. Therefore, adaptive management strategies that consider uncertain future scenarios are vital for ensuring the resilience of *C. hispidus* to climate change across its distribution range.

Given its endangered status and the lack of ecological studies, the present study aims to represent the first SDM conducted for the hispid hare. This approach was adopted to identify potential habitats in both current and future scenarios, aiming to prioritize and guide conservation strategies in its distribution range and protected areas. It will further aid in adaptive management and provide valuable insights into suitable habitats, which could serve as potential translocation sites for the species.

## 2. Materials and Methods

### 2.1. Study Area and Species Occurrence Records (SORs)

**Figure 1:**
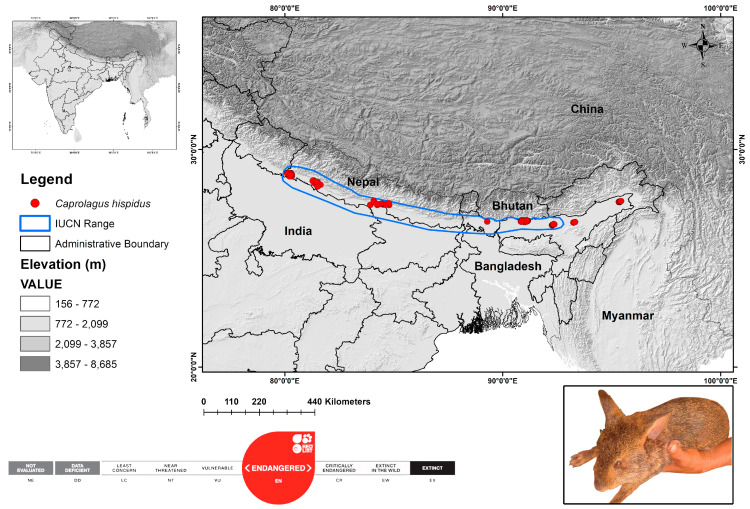
The map illustrates the global range distribution and observed locations of endangered hispid hare, *C. hispidus*. Color code represents the elevation gradient in the study landscape. The original image of hispid hare reproduced with permission through direct communication with the original copyright holder Bhaskar Choudhury.

Historically, the hispid hare inhabited the southern foothills of the Himalayas, ranging from Uttar Pradesh to Assam in India and Nepal. Currently, the hispid hare’s distribution in southern Asia is patchy, encompassing Nepal, Bhutan, Bangladesh, and India, with elevations typically ranging from 100 to 250 m [5,11,17]. Thus, for the present study, the geographical range was considered as the study area that encompasses the historical range as well as the range assessed by the IUCN Red List specialist group (Figure 1). This scientific study utilized data from secondary sources from the platform Geospatial Conservation Assessment Tool (GeoCat) accessed on 15 February 2024 [41], which synchronizes with GBIF (*n* = 66) [42] and iNaturalist (*n* = 6) (https://www.inaturalist.org/) along with information extracted from peer-reviewed scientific literature (*n* = 30) [5,6,10,11,14,17,18,23,25,41,43]. A total of 102 identified locations of the targeted species were accumulated for the study.

### 2.2. Model Covariates

Considering the ecological requirements of *C. hispidus*, the covariates included climatic, habitat, anthropogenic, and topographic variables that could potentially affect the prediction of suitable habitats. The climatic factors, specifically the 19 standard bioclimatic variables were sourced from Worldclim, Version 2.0 [44]. Additionally, the analysis also incorporated three habitat variables, namely Normalized Difference Vegetation Index (NDVI), shrublands (euc_20), and herbaceous wetlands (euc_90) based on the IUCN assessment [16]. The anthropogenic variable builtup/urban (euc_50) was also taken into account to analyze its impact on the species as per IUCN assessment [16]. The variables shrubland, herbaceous wetland and builtup/urban taken for the final MaxEnt model were computed utilizing the Euclidean distance function in ArcGIS 10.6 from the Land Use and Land Cover (LULC) classes data. All the habitat variables were obtained from the Copernicus Global Land Service [45,46,47]. Topographic variables, such as elevation and aspect, were derived from the 90 m Shuttle Radar Topography Mission (SRTM) data, accessible online [37,46]. All predictors underwent resampling to a spatial resolution of 1 km^2^ using the spatial analysis tool within ArcGIS 10.6. Spatial correlation among the predictors was assessed using SDM Toolbox v2.4, and variables showing a correlation coefficient r > 0.8 were excluded from the final model (Figure S1) [48,49]. Furthermore, to project climate change scenarios under three distinct Shared Socio-economic Pathways (SSP)—namely ssp126, ssp245, and ssp585—for the periods 2041–2060 and 2061–2080, this study utilized the General Circulation Model (GCM) Hadley Centre Global Environment Model in the Global Coupled Configuration 3.1 (HadGEM3-GC31 LL) as part of the UK’s contribution to the sixth Coupled Model Intercomparison Project (CMIP6), as it is one of the best performing model in the South and Southeast Asia [50,51]. Furthermore, this GCM doesn’t encounter difficulties in capturing temporal fluctuations and excels more in depicting temperature distribution, especially when analyzing specific homogeneous temperature regions. Notably, non-climatic raster data (elevation, aspect, slope, ndvi, shrubland, herbaceous wetland, and built-up) remained constant for this analysis to evaluate the isolated effect of climate change on the study objectives. This was done in order to restrict the distribution probabilities in the possible habitat zones within the study range and to eliminate projections regions such as permafrost and barren plateau areas. 

### 2.3. Model Development

The modeling software utilized was MaxEnt Ver. 3.4.4, well-known for its robust performance in predicting species distribution models [52,53]. Model development incorporated the bootstrapping replication approach and the Bernoulli generalized linear model with the ClogLog link function [54]. In this process, training data for each occurrence point were treated as n − 1, and model execution was assessed, with residual points over 20 runs as replicates [55]. The spatial jackknife test of acquired regularized training gain determined variable influence on occurrences [52]. Model evaluation relied on the area under the curve statistics (AUC) of the receiver operating characteristic (ROC) curve [56]. AUC test values ranging from 0 to 1 were interpreted, where values below 0.5 indicated deficient power, 0.5 suggested random prediction, 0.7–0.8 were considered acceptable, 0.8–0.9 deemed excellent, and >0.9 regarded as exceptional model performance [40,57,58]. Furthermore, the true skill statistic (TSS) score for the present model was assessed for validation [59]. Binary maps were generated using an equal test sensitivity and specificity (SES) threshold for predicting suitable habitat for the targeted species, and the raster calculator was employed to evaluate zonal statistics through the Zonal Statistics Tool in ArcGIS 10.6 [46].

### 2.4. Habitat Quality Assessment

Comparative analyses were performed on suitable areas of *C. hispidus* using both present and future climatic models. Class-level metrics, such as the number of patches (NP), aggregate index (AI), patch density (PD), largest patch index (LPI), edge density (ED), total edge (TE), and landscape shape index (LSI), were calculated using FRAGSTATS version 4.2.1 [60]. These metrics are especially valuable for delineating ecological processes, as they offer insights into how alterations in suitable areas impact landscape dynamics. This allows for a more detailed characterization of landscape attributes and enables a comprehensive analysis throughout the distribution range of the species [61]. They were utilized as indicators to assess habitat characteristics and the degree of fragmentation in the modeled area under current and climate change scenarios [40]. A qualitative evaluation of the habitat ranges was conducted by calculating zonal statistics within the boundaries of protected areas (PAs) across their distribution range in present and future scenarios [62].

## 3. Results

### 3.1. Species Distribution Model

**Figure 2:**
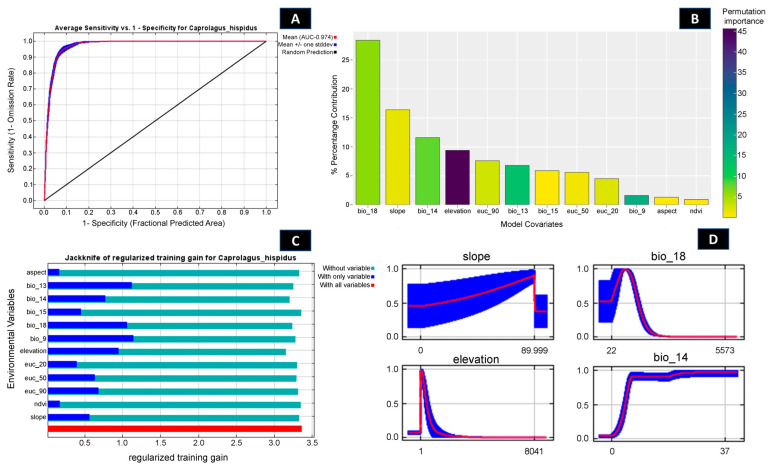
Showing model evaluation along with variable influence. (**A**) The average training ROC (Receiver Operating Characteristics) for the model. (**B**) Percentage contribution and permutation importance of covariates. (**C**) Jackknife test for all the selected variables, where the blue bar shows the importance of each variable in explaining the data variation when used separately. The green bar shows the loss in overall gain after the particular variable was dropped. Red bar = total model gain. (**D**) The response curves of the major contributing predictors governing the habitat suitability of *C. hispidus*.

**Figure 3:**
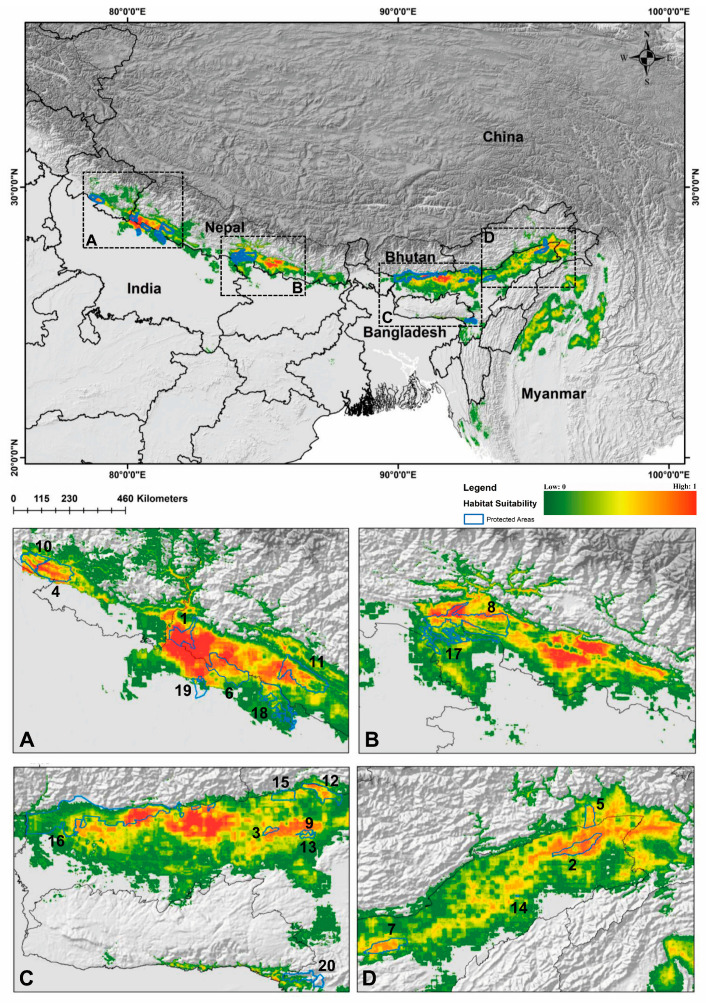
Map representing the present suitable habitat for hispid hare, *C. hispidus* in the distribution range and protected areas. The subfigures (**A**–**D**) illustrate the partial enlargements of above map, intended to demonstrate the habitat quality of the Protected Areas (PAs). 1. Shuklaphanta National Park, 2. Dibru-Saikhowa National Park, 3. Orang National Park, 4. Corbett National Park, 5. D’Ering Memorial Wildlife Sanctuary, 6. Dudhwa National Park, 7. Kaziranga National Park, 8. Chitawan National Park, 9. Burachapori Wildlife Sanctuary, 10. Sonanandi Wildlife Sanctuary, 11. Bardia National Park, 12. Nameri National Park, 13. Laokhowa Wildlife Sanctuary, 14. Pani-Dihing Wildlife Sanctuary, 15. Sonai-Rupai Wildlife Sanctuary, 16. Manas Tiger Reserve, 17. Valmiki National Park, 18. Katerniaghat Wildlife Sanctuary, 19. Kishanpur Wildlife Sanctuary, and 20. Borail Wildlife Sanctuary.

Out of 102 identified locations, 97 points were screened through autocorrelation using the spatial rarefy occurrence point function in SDM Toolbox v2.4 and selected for the final model. The results revealed that, on average, the training area under the curve (AUC) over multiple runs was 0.974, with a standard deviation (SD) of 0.001 (Figure 2 and Figures S2–S4) and the TSS score was found to be 0.8641. Within the total geographical extent of 188,316 km^2^, approximately 11,374 km^2^ (6.03%) were identified as highly suitable areas for *C. hispidus* (Figure 3). Additionally, the analysis highlighted that among the bioclimatic variables, the Mean Temperature of Wettest Quarter (Bio_18) contributed the most (28.4%) to the model, while the anthropogenic variable, Euclidean Distance to Urban/built up (euc_50), contributed 5.6% to the model. Among the habitat variables, Euclidean Distance to Herbaceous wetland (euc_90) contributed the most (7.6%) to the model, while the NDVI had the lowest contribution (0.9%). Within the topographic variables, slope contributed 16.4% to the model and was the second highest contributor to the model (Figure 2 and Figure 3; Table S1). Moreover, the topographic variable’s elevation was found to have a permutation importance of 45.6%, suggesting its influence in demarcating a suitable habitat for the species (Figure 2).

**Figure 4:**
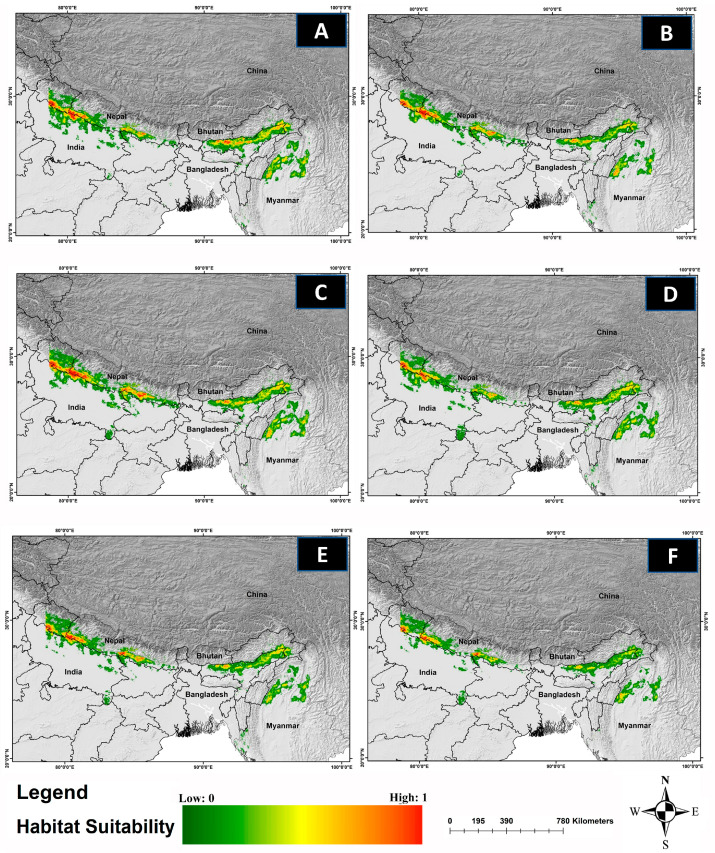
The habitat suitability for *C. hispidus* in future climatic projection scenarios of ssp126, ssp245, and ssp585 for the years 2041–2060 and 2061–2080. The projection for the years: (**A**) 2041–2060-SSP-126, (**B**) 2061–2080-SSP-126, (**C**) 2041–2060-SSP-245, and (**D**) 2061–2080-SSP-245, (**E**) 2041–2060-SSP-585, (**F**) 2061–2080-SSP-585.

The comparative analysis of current and future models reveals a significant reduction in the suitable habitat for *C. hispidus* in upcoming scenarios. Specifically, from 2041 to 2060, the anticipated decrease is projected to be around 6.62% for ssp126, 8.06% for ssp245, and 27.53% for ssp585, compared with the existing distribution (11,374 km^2^). Additionally, for the timeframe spanning 2061 to 2080, the findings indicate a decline of 12.35% for ssp126, 52.02% for ssp245, and 62.27% for ssp585 (Figure 4; Table S2).

### 3.2. Habitat Quality, Geometry, and Complexity

**Figure 5:**
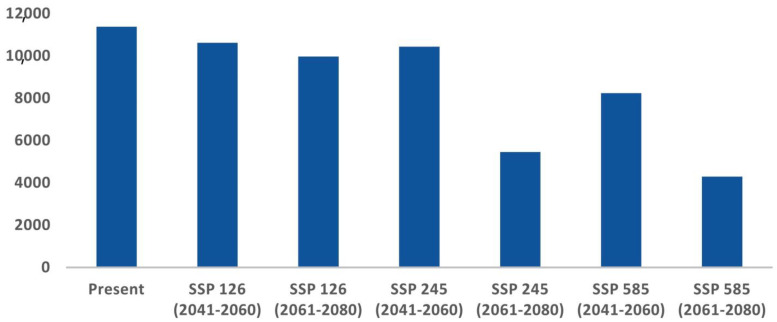
Habitat quality assessment for *C. hispidus* in present and future scenarios: Area (km^2^) change trend from present to future scenarios.

In the current situation, elevated levels of NP (415), PD (2014489.8811), TE (67.8080), LPI (0.1116), and LSI (19.8178) suggest a high abundance of larger patches with complex geometric shapes, while the low value of AI (82.1292) indicates that these habitat patches are relatively distant from each other (Figure 5; Table 1). However, in the future projections across the SSP126, SSP245, and SSP585, it was observed that NP, PD, TE, LPI, LSI, and ED have decreased, indicating the impact of climate change. This suggests that in the future, habitat patch areas will be fewer and smaller and exhibit simpler geometry compared with the present situation. Additionally, in some instances of higher AI values, it was noted that these patches are closer together compared with the current scenario (Figure 5; Table 1).

An analysis of the fragmentation metrics revealed a decline in NP and LPI during the time period 2041–2060 for SSP126, SSP245, and SSP585 compared with the present scenario. Specifically, NP decreased by 18.07%, 3.37%, and 29.37%, respectively, while LPI decreased by 9.05%, 5.46%, and 19.17%, respectively. However, the situation worsened during the period 2061–2080, with NP decreasing by 22.65%, 44.81%, and 55.66%, respectively, and LPI decreasing by 34.40%, 44.89%, and 62.36%, respectively, compared with the present scenario (Figure 5; Table 1). These declines of NP and LPI in the future change scenarios explained that habitat patches have reduced in numbers as well as being smaller in size. Due to reductions in NP and LPI, indicating fewer and smaller patch sizes due to habitat loss, other metrics such as PD, TE, and ED in future climate scenarios have been significantly impacted throughout the distribution range. These metrics are heavily dependent on NP and LPI and consequently experience substantial reductions. For instance, PD exhibited a decline of up to 55.66% in the SSP585 (2061–2080) scenario, indicating a decrease in density of the patches corresponding to the reduced NP. Similarly, TE decreased by varying percentages, ranging from 2.62% to 61.09%, reflecting a decline in edge area for patches. Additionally, ED decreased by 61% in the SSP585 (2061–2080), suggesting a decrease in density of the edges resulting from changes in TE, NP, and LPI compared with the present scenario. Moreover, the measure of patch shape geometry, LSI, decreased by up to 36.39% in the SSP585 (2061–2080) compared with the present scenario. Furthermore, AI, representing patch proximity, increased in all future scenarios, with a maximum increase of 4.33% in the SSP245 (2041–2060) compared with the present. However, there was a slight decline of 0.3% in the SSP585 (2061–2080), possibly due to rapid declines in metrics such as NP, LPI, and LSI in this particular scenario. Overall, these metrics were directly influenced by the decline in habitat, in conjunction with reductions in NP and LPI. They indicate that in future climate scenarios, habitat patches will be fewer in number, smaller in size, and in close proximity, compared with the present scenario, suggesting habitat fragmentation in suitable areas of *C. hispidus* (Table 1).

### 3.3. Representativeness of the Protected Area for Conservation

The habitat presence within Protected Areas (PAs) across the distribution range of *C. hispidus* was assessed (Table S3). The top 20 PAs with the greatest extent of habitat within the species study range were identified (Table 2). Among these, Shuklaphanta National Park (ShNP) (0.836) in the Mahakali Province of Nepal exhibited the highest mean extent of habitat, followed by Dibru-Saikhowa National Park (DSNP) (0.631) in Assam, India. DSNP also emerged as the highest suitable habitat for the *C. hispidus* in India, followed by Orang National Park (ONP) (0.572). Among the suitable protected areas spanning transboundary countries within its distribution range, it was noted that three protected areas, namely Katerniaghat Wildlife Sanctuary (KgWLS) (0.191) and Kishanpur Wildlife Sanctuary (KWLS) (0.108) in the state of Uttar Pradesh and Borail Wildlife Sanctuary (0.102) in Assam, India, had the least mean suitable area among the identified top 20 PAs (Table 2).

In the scenario of SSP126, during the time periods of 2041–2060 and 2061–2080, it was observed that ShNP experienced a decrease in mean habitat extent by 1.2% and 2.42%, respectively. Similarly, DSNP also exhibited a decline of 23.22% and 38.51%, respectively. However, Corbett National Park (CNP) demonstrated an increase in mean habitat extent of 43.14% and 28.45%, respectively, while Sonanandi Wildlife Sanctuary (SWLS) also showed an increase in mean habitat suitability of 71.59% and 40.12%, respectively (Table 2).

Whereas, in the SSP245 scenario, spanning the time periods of 2041–2060 and 2061–2080, ShNP witnessed an increase in mean habitat of 7.28% and 6.17%, respectively. Conversely, DSNP experienced declines of 32.30% and 46.20%, respectively. The CNP demonstrated an increase in mean habitat of 45.66% and 13.25%, respectively, and SWLS also exhibited increases of 52.62% and 33.41%, respectively. Interestingly, during the period of 2041–2060, Dudhwa National Park (DNP) and Chitawan National Park (ChNP) saw increases of 36.57% and 17.86%, respectively. However, these trends reversed during the period of 2061–2080, with declines of 45.75% and 62.40%, respectively, compared with the present scenario.

Again, in the SSP585 scenario, spanning the time periods of 2041–2060 and 2061–2080, ShNP saw declines in mean habitat by 10.52% and 29.88%, respectively. Similarly, DSNP also experienced declines of 40.19% and 65.72%, respectively. However, notably, CNP and SWLS demonstrated an increase in mean habitat of 10.65% and 42.38%, respectively, during the time periods of 2041–2060 compared with the present scenario. However, these areas witnessed declines of 9.72% and 5.70%, respectively, during the time periods of 2061–2080 compared with the current scenario (Table 2).

## 4. Discussion

Acquiring insights into the spatial utilization patterns of *C. hispidus* holds significance for efficient wildlife management and the formulation of conservation planning strategies for this species, as well as for other grassland species such as the Bengal Florican, *Houbaropsis bengalensis* [5,43]. The present study revealed concerning trends regarding the species’ habitat extent across its entire geographical range. While only around 6.03% of the total area was identified as habitat, the analysis indicates a significant reduction in habitat areas under various climate change scenarios (Figure 3). Projections for 2041 to 2060, across different socioeconomic pathways, indicate alarming declines in habitat. Even under more optimistic scenarios (SSP126), there is a substantial loss of habitat, with projected declines of 6.62%. More severe scenarios (SSP245 and SSP585) show even greater reductions, with declines of 8.06% and 27.53%, respectively (Figure 4). These findings underscore the significant threats the species faces, particularly under more severe emissions scenarios. Looking further ahead, projections for 2061 to 2080 paint a grim picture, with even more pronounced decreases in habitat availability. Anticipated declines of 12.35% for SSP126, 52.02% for SSP245, and 62.27% for SSP585 highlight the escalating impacts of climate change. Particularly under severe emissions scenarios (SSP585), there is a risk of drastic habitat loss and fragmentation, posing existential threats to the *C. hispidus* population.

The projected decline in habitat for *C. hispidus* can be attributed to several factors. Climate change-induced shifts in temperature and precipitation patterns are expected to directly influence vegetation cover and the availability of suitable food resources for the species. Additionally, human-induced alterations in land use and habitat degradation further contribute to the loss and fragmentation of habitats [5], with local human populations utilizing grasslands for agricultural and domestic purposes, leading to a reduction in habitats for grassland-dependent wildlife, including the *C. hispidus* [63]. Studies in various parks have highlighted habitat loss and fragmentation as significant threats [6,14,17,25,64]. The conversion of grasslands into woodlands poses another severe threat to *C. hispidus* existence [25,65]. This natural succession reduces essential grass vegetation, impacting the food and cover requirements of the species [16]. The presence of *C. hispidus* is confined to isolated patches of grassland within national parks, and its population is rapidly declining due to anthropogenic pressure and grassland fires across its range [11]. Seasonal burning practices to stop succession, overlapping with the breeding season of the *C. hispidus*, could potentially have negative impacts on the species’ survival [10,17]. Therefore, implementing alternate grassland burning practices that avoid coinciding with the breeding season could be preferable to ensure the species’ survival and reproduction [10]. The findings from the analysis of habitat fragmentation underscore the significant effects of future climate change scenarios on landscape dynamics and habitat fragmentation for the species. The decreases observed in both NP and LPI indicate a troubling trend of diminishing habitat patches, which could have adverse consequences for the species. Moreover, the notable reductions in PD, TE, and ED highlight the severity of habitat fragmentation, suggesting a decline in patch density and the availability of edge habitats. Furthermore, the decrease in patch shape complexity, as indicated by the decline in LSI, suggests a decrease in habitat complexity. Additionally, the observed increase in AI in most future scenarios implies a greater clustering of habitat patches. The variability in fragmentation metrics across different future climate scenarios underscores the complexity of predicting habitat dynamics and the importance of considering multiple scenarios in conservation planning. Furthermore, the disproportionate impacts observed in the SSP585 (2061–2080) scenario highlight the urgency of addressing factors driving habitat loss and fragmentation, particularly in regions where future projections indicate significant declines in suitable habitat (Figure 5; Table 1). This worsening trend in fragmentation underscores the escalating impacts of climate change on habitat suitability for *C. hispidus* [46].

The assessment of mean habitat extent within PAs across the distribution range of *C. hispidus* provides crucial insights into the conservation status of this species and the potential impacts of climate change on its habitat. According to previous studies, historically, the *C. hispidus* was believed to inhabit three protected areas in Nepal (ShNP, BNP, and ChNP). However, since the 1980s, sightings have been reported only from BNP and ShNP [63], leading to their presumed extinction in ChNP [11]. In a targeted survey for grassland birds, an individual hispid hare was rediscovered in the Sukhibhar grassland in the ChNP on 30 January 2016, marking its presence in the ChNP after several decades [5,11]. This sighting revived its presence in ChNP, marking the first photographic confirmation of its presence since 1984 [11]. In India, the hispid hare has been confirmed in protected areas such as Jaldapara Wildlife Sanctuary (JWLS), BaWLS, MNP, and DNP within its distribution range [5,14,18,43]. Among these areas, 20 PAs were identified as having the highest availability for the hispid hare, with ShNP in Nepal and DSNP in India emerging as key habitats. Interestingly, JWLS and BaWLS exhibited lower mean habitat availability and were therefore excluded from the top 20 list, possibly due to fragmented grasslands dominated by *Saccharum narenga* and *Arundo donax* [43]. In JWLS, efforts by the Forest Department to plant palatable grass species primarily for camp elephants have resulted in patches insufficient to support for *C. hispidus* in terms of food and shelter. Large open spaces between grass clumps act as barriers or deterrents to hare movement, particularly during grassland burning seasons [43]. It is noteworthy that two protected areas in Uttarakhand, specifically CNP and SWLS, have demonstrated high mean suitability for *C. hispidus*. However, there have been no reported sightings of this species in these areas to date. This assessment suggests the need for further research in these two protected areas, as the species may be present but has potentially been overlooked thus far. Considering that these protected areas exhibit an increase in habitat in the future, they could also be considered potential translocation sites for the species (Table 2).

In Nepal, previous studies suggested that ShNP has areas that are less suitable for grassland species such as greater one-horned rhinoceros, also sharing similar feeding preferences with the hispid hare compared with other protected areas [66,67]. However, the current study contradicts this assessment by revealing that ShNP exhibits the highest mean habitat extent for *C. hispidus* among all the other protected areas assessed.

In Bhutan, historical evidence, anecdotal sources, and sign surveys indicated the potential presence of the hispid hare along the southern foothills. Consequently, a study was conducted to assess the presence of *C. hispidus* in Royal Manas National Park (RMNP), resulting in the capture of photographic evidence using camera traps [25]. However, the present study indicates RMNP exhibits very low mean habitat extent for *C. hispidus*, leading to its exclusion from the final list of top 20 protected areas. Instead, the Indian counterpart, MNP, demonstrates relatively higher suitability compared with its Bhutanese counterpart. Therefore, adaptive management strategies that account for uncertain future scenarios are essential for ensuring the resilience of *C. hispidus* to climate change in its distribution range.

## 5. Conclusions

In conclusion, this study investigated the distribution pattern of *C. hispidus* using species distribution modelling for the first time and underscores the critical need for conservation and further research on this species. The findings also revealed concerning trends regarding habitat availability across the species’ geographical range, with projected declines under various climate change scenarios. The anticipated reductions in habitat, particularly under severe emissions scenarios, highlight the significant threats faced by *C. hispidus.* Conservation efforts should prioritize protecting key habitats within the PAs and implementing adaptive management strategies. The assessment of habitat within PAs provides crucial insights into the conservation status of the species. Although certain PAs such as ShNP, DSNP, and ONP were identified as key habitats, others such as CNP and SWLS showed high suitability in both present and future scenarios despite no recorded sightings within these PAs. This underscores the future research prospects associated with these PAs and their potential as translocation sites for implementing adaptive management strategies to ensure the resilience of *C. hispidus* to climate change. Therefore, transboundary cooperation and coordinated conservation initiatives are vital for ensuring the species’ long-term viability across its range. Continuous monitoring and adaptive management based on updated climate projections are essential to guide effective conservation actions and secure the future of *C. hispidus*. The implications of these findings are significant for *C. hispidus* conservation. Declining suitable habitat areas increases the species’ vulnerability to population decline, range reduction, and local extinctions. Conservation strategies should prioritize habitat protection, restoration, and connectivity to counteract habitat loss and fragmentation. Adaptive management strategies, considering uncertain future scenarios, are crucial to enhancing the species’ resilience to climate change. Collaboration among researchers, policymakers, and stakeholders is pivotal for implementing effective conservation measures that address present and future challenges. In summary, proactive conservation actions are urgently needed to safeguard *C. hispidus* habitat amid ongoing and projected climate change. By addressing the drivers of habitat loss and fragmentation, efforts can be directed toward ensuring the long-term survival and persistence of this endangered species.

## Figures and Tables

**Table 1:** Habitat quality assessment of *C. hispidus* in present and future scenarios. NP: No. of Patches, PD: Patch Density, LPI: Largest Patch Index, ED: Edge Density, TE: Total Edge, LSI: Landscape Shape Index; AI: Aggregation Index.

Scenarios	NP	PD	LPI	TE	ED	LSI	AI
Present	415	2,014,490	0.1116	67.808	3291.53	19.8178	82.1292
SSP 126 (2041–2060)	340	1,650,425	0.1115	55.408	2689.61	16.8309	84.4197
SSP 126 (2061–2080)	321	1,558,196	0.0732	55.848	2710.97	17.53	83.2506
SSP 245 (2041–2060)	401	1,946,531	0.1055	69.184	3358.32	18.029	85.6869
SSP 245 (2061–2080)	229	1,111,610	0.0615	32.176	1561.89	13.6959	82.5469
SSP 585 (2041–2060)	293	1,422,278	0.0902	46.656	2264.77	16.1209	83.1186
SSP 585 (2061–2080)	184	893,171	0.042	26.384	1280.73	12.6061	81.8698

**Table 2:** Mean suitability of top 20 protected areas in present and future scenarios. ShNP: Shuklaphanta National Park; DSNP: Dibru-Saikhowa National Park; ONP: Orang National Park; CNP: Corbett National Park; DMWLS: D’Ering Memorial Wildlife Sanctuary; DNP: Dudhwa National Park; KNP: Kaziranga National Park; ChNP: Chitawan National Park; BWLS: Burachapori Wildlife Sanctuary; SWLS: Sonanandi Wildlife Sanctuary; BNP: Bardia National Park; NNP: Nameri National Park; LWLS: Laokhowa Wildlife Sanctuary; PDWLS: Pani-Dihing Wildlife Sanctuary; SRWLS: Sonai-Rupai Wildlife Sanctuary; MTR: Manas Tiger Reserve; VNP: Valmiki National Park; KgWLS: Katerniaghat Wildlife Sanctuary; KWLS: Kishanpur Wildlife Sanctuary; BoRWLS: Borail Wildlife Sanctuary.

Sl. No.	Country	State/Province	Protected Area	Mean Suitability (Present)	SSP126 (2041–2060)	SSP126 (2061–2080)	SSP245 (2041–2060)	SSP245 (2061–2080)	SSP585 (2041–2060)	SSP585 (2061–2080)
1	Nepal	Mahakali Province	ShNP	0.837	0.827	0.816	0.898	0.888	0.749	0.587
2	India	Assam	DSNP	0.631	0.485	0.388	0.427	0.340	0.378	0.216
3	India	Assam	ONP	0.572	0.491	0.425	0.427	0.239	0.231	0.213
4	India	Uttarakhand	CNP	0.530	0.759	0.681	0.772	0.601	0.587	0.479
5	India	Arunachal Pradesh	DMWLS	0.477	0.389	0.235	0.340	0.272	0.282	0.163
6	India	Uttar Pradesh	DNP	0.464	0.478	0.496	0.634	0.252	0.344	0.209
7	India	Assam	KNP	0.463	0.495	0.199	0.343	0.266	0.280	0.175
8	Nepal	Bagmati Province	ChNP	0.446	0.297	0.353	0.526	0.168	0.404	0.301
9	India	Assam	BWLS	0.437	0.407	0.351	0.349	0.190	0.191	0.170
10	India	Uttarakhand	SWLS	0.423	0.726	0.593	0.646	0.565	0.603	0.399
11	Nepal	Lumbini Province	BNP	0.384	0.330	0.387	0.485	0.175	0.352	0.213
12	India	Assam	NNP	0.376	0.336	0.171	0.201	0.077	0.123	0.091
13	India	Assam	LWLS	0.329	0.311	0.237	0.276	0.143	0.156	0.126
14	India	Assam	PDWLS	0.318	0.333	0.164	0.276	0.227	0.245	0.151
15	India	Assam	SRWLS	0.266	0.172	0.103	0.115	0.046	0.044	0.047
16	India	Assam	MTR	0.245	0.133	0.077	0.096	0.064	0.079	0.029
17	India	Bihar	VNP	0.216	0.067	0.120	0.357	0.088	0.208	0.120
18	India	Uttar Pradesh	KgWLS	0.192	0.211	0.209	0.270	0.095	0.101	0.096
19	India	Uttar Pradesh	KWLS	0.108	0.716	0.539	0.407	0.513	0.228	0.138
20	India	Assam	BoRWLS	0.103	0.105	0.106	0.102	0.103	0.102	0.102

## Data Availability

Data used for the analysis were sourced from open-access resources.

## Supplementary Materials

The following supporting information can be downloaded at: https://www.mdpi.com/article/10.3390/biology13030198/s1, Figure S1. Correlation metrics of covariates represents the spatial correlation among the predictors assessed using SDM Toolbox v2.4; Figure S2. The training omission rate and predicted area as a function of the cumulative threshold, averaged over the 20 replicate runs; Figure S3. (A) The image shows the jackknife test, using AUC on test data. (B) The image shows the jackknife test of test gain; Figure S4. The curves show how each environmental variable affects the model prediction and how the predicted probability of presence changes as each environmental variable is varied, keeping all other environmental variables at their average sample value. It also shows the mean response of the 20 replicate MaxEnt runs (red) and the mean ± one standard deviation (blue, two shades for categorical variables); Table S1. Percentage contribution and permutation contribution with covariates details; Table S2. Estimated suitable habitat (in km^2^) in different climate change scenarios; Table S3. Protected Areas in the distribution range of *C. hispidus*, out of which, the top 20 are demonstrated in Table 1. NP: National Park; WLS: Wildlife Sanctuary.
