# Supplementary material for: Distribution Model Reveals Rapid Decline in Habitat Extent for Endangered Hispid Hare: Implications for Wildlife Management and Conservation Planning in Future Climate Change Scenarios
Source: Biology (Basel). 2024 Mar 20;13(3):198. doi: 10.3390/biology13030198 (PMC10967808; doi:10.3390/biology13030198)
Supplement: Supplementary file 1 [file biology-13-00198-s001.zip › biology-2900517-supplementary.pdf]

Supplementary Materials

Covariates	aspect	bio_1	bio_10	bio_11	bio_12	bio_13	bio_14	bio_15	bio_16	bio_17	bio_18	bio_19	bio_2	bio_3	bio_4	bio_5	bio_6	bio_7	bio_8	bio_9	elevation	euc_20	euc_50	euc_90	ndvi	slope
aspect	1.000	-0.025	-0.029	-0.022	0.022	0.015	0.045	-0.030	0.015	0.031	0.036	0.023	-0.006	0.033	-0.005	-0.033	-0.021	-0.018	-0.024	-0.027	0.023	0.008	0.014	0.013	0.007	0.116
bio_1	-0.025	1.000	0.991	0.996	0.636	0.673	0.046	0.436	0.681	0.272	0.319	0.007	-0.457	0.289	-0.761	0.966	0.991	-0.514	0.967	0.952	-0.993	-0.335	-0.643	-0.488	0.375	-0.058
bio_10	-0.029	0.991	1.000	0.977	0.568	0.631	-0.003	0.508	0.633	0.221	0.240	-0.022	-0.371	0.197	-0.672	0.989	0.968	-0.401	0.964	0.952	-0.981	-0.306	-0.610	-0.469	0.361	-0.059
bio_11	-0.022	0.996	0.977	1.000	0.666	0.690	0.073	0.392	0.701	0.294	0.350	0.021	-0.502	0.340	-0.815	0.946	0.998	-0.575	0.955	0.944	-0.989	-0.359	-0.655	-0.496	0.375	-0.053
bio_12	0.022	0.636	0.568	0.666	1.000	0.957	0.451	0.087	0.974	0.542	0.876	0.219	-0.751	0.408	-0.756	0.470	0.690	-0.798	0.629	0.553	-0.669	-0.237	-0.456	-0.319	0.286	-0.023
bio_13	0.015	0.673	0.631	0.690	0.957	1.000	0.354	0.299	0.993	0.470	0.791	0.182	-0.646	0.297	-0.675	0.557	0.710	-0.663	0.665	0.607	-0.697	-0.260	-0.471	-0.344	0.275	-0.024
bio_14	0.045	0.046	-0.003	0.073	0.451	0.354	1.000	-0.307	0.367	0.856	0.529	0.689	-0.468	0.141	-0.258	-0.078	0.108	-0.455	0.057	0.022	-0.081	-0.160	-0.123	-0.032	-0.012	0.024
bio_15	-0.030	0.436	0.508	0.392	0.087	0.299	-0.307	1.000	0.263	-0.222	-0.122	-0.295	0.105	-0.320	0.007	0.558	0.373	0.232	0.490	0.411	-0.423	-0.104	-0.319	-0.263	0.138	-0.024
bio_16	0.015	0.681	0.633	0.701	0.974	0.993	0.367	0.263	1.000	0.476	0.805	0.171	-0.680	0.321	-0.706	0.552	0.721	-0.702	0.673	0.608	-0.706	-0.256	-0.476	-0.346	0.289	-0.025
bio_17	0.031	0.272	0.221	0.294	0.542	0.470	0.856	-0.222	0.476	1.000	0.524	0.819	-0.529	0.221	-0.405	0.152	0.331	-0.544	0.261	0.254	-0.300	-0.219	-0.259	-0.134	0.027	-0.001
bio_18	0.036	0.319	0.240	0.350	0.876	0.791	0.529	-0.122	0.805	0.524	1.000	0.231	-0.664	0.421	-0.539	0.125	0.378	-0.728	0.349	0.225	-0.373	-0.103	-0.263	-0.163	0.206	-0.007
bio_19	0.023	0.007	-0.022	0.021	0.219	0.182	0.689	-0.295	0.171	0.819	0.231	1.000	-0.247	0.086	-0.124	-0.050	0.055	-0.255	-0.053	0.115	-0.018	-0.144	-0.075	0.000	-0.123	0.012
bio_2	-0.006	-0.457	-0.371	-0.502	-0.751	-0.646	-0.468	0.105	-0.680	-0.529	-0.664	-0.247	1.000	-0.148	0.722	-0.256	-0.549	0.895	-0.455	-0.363	0.496	0.244	0.381	0.281	-0.155	0.047
bio_3	0.033	0.289	0.197	0.340	0.408	0.297	0.141	-0.320	0.321	0.221	0.421	0.086	-0.148	1.000	-0.634	0.142	0.332	-0.568	0.215	0.265	-0.279	-0.197	-0.291	-0.161	0.227	0.035
bio_4	-0.005	-0.761	-0.672	-0.815	-0.756	-0.675	-0.258	0.007	-0.706	-0.405	-0.539	-0.124	0.722	-0.634	1.000	-0.596	-0.828	0.891	-0.688	-0.691	0.759	0.428	0.616	0.448	-0.315	0.020
bio_5	-0.033	0.966	0.989	0.946	0.470	0.557	-0.078	0.558	0.552	0.152	0.125	-0.050	-0.256	0.142	-0.596	1.000	0.932	-0.282	0.935	0.942	-0.948	-0.294	-0.579	-0.449	0.341	-0.053
bio_6	-0.021	0.991	0.968	0.998	0.690	0.710	0.108	0.373	0.721	0.331	0.378	0.055	-0.549	0.332	-0.828	0.932	1.000	-0.611	0.951	0.939	-0.986	-0.362	-0.650	-0.491	0.367	-0.053
bio_7	-0.018	-0.514	-0.401	-0.575	-0.798	-0.663	-0.455	0.232	-0.702	-0.544	-0.728	-0.255	0.895	-0.568	0.891	-0.282	-0.611	1.000	-0.475	-0.427	0.541	0.318	0.456	0.320	-0.226	0.024
bio_8	-0.024	0.967	0.964	0.955	0.629	0.665	0.057	0.490	0.673	0.261	0.349	-0.053	-0.455	0.215	-0.688	0.935	0.951	-0.475	1.000	0.889	-0.972	-0.289	-0.598	-0.456	0.379	-0.061
bio_9	-0.027	0.952	0.952	0.944	0.553	0.607	0.022	0.411	0.608	0.254	0.225	0.115	-0.363	0.265	-0.691	0.942	0.939	-0.427	0.889	1.000	-0.938	-0.341	-0.609	-0.451	0.323	-0.056
elevation	0.023	-0.993	-0.981	-0.989	-0.669	-0.697	-0.081	-0.423	-0.706	-0.300	-0.373	-0.018	0.496	-0.279	0.759	-0.948	-0.986	0.541	-0.972	-0.938	1.000	0.327	0.646	0.483	-0.384	0.061
euc_20	0.008	-0.335	-0.306	-0.359	-0.237	-0.260	-0.160	-0.104	-0.256	-0.219	-0.103	-0.144	0.244	-0.197	0.428	-0.294	-0.362	0.318	-0.289	-0.341	0.327	1.000	0.557	0.616	-0.138	-0.001
euc_50	0.014	-0.643	-0.610	-0.655	-0.456	-0.471	-0.123	-0.319	-0.476	-0.259	-0.263	-0.075	0.381	-0.291	0.616	-0.579	-0.650	0.456	-0.598	-0.609	0.646	0.557	1.000	0.647	-0.279	0.019
euc_90	0.013	-0.488	-0.469	-0.496	-0.319	-0.344	-0.032	-0.263	-0.346	-0.134	-0.163	0.000	0.281	-0.161	0.448	-0.449	-0.491	0.320	-0.456	-0.451	0.483	0.616	0.647	1.000	-0.198	0.024
ndvi	0.007	0.375	0.361	0.375	0.286	0.275	-0.012	0.138	0.289	0.027	0.206	-0.123	-0.155	0.227	-0.315	0.341	0.367	-0.226	0.379	0.323	-0.384	-0.138	-0.279	-0.198	1.000	0.003
slope	0.116	-0.058	-0.059	-0.053	-0.023	-0.024	0.024	-0.024	-0.025	-0.001	-0.007	0.012	0.047	0.035	0.020	-0.053	-0.053	0.024	-0.061	-0.056	0.061	-0.001	0.019	0.024	0.003	1.000

Figure S1. Correlation metrics of covariates represents the spatial correlation among the predictors assessed using SDM Toolbox v2.4

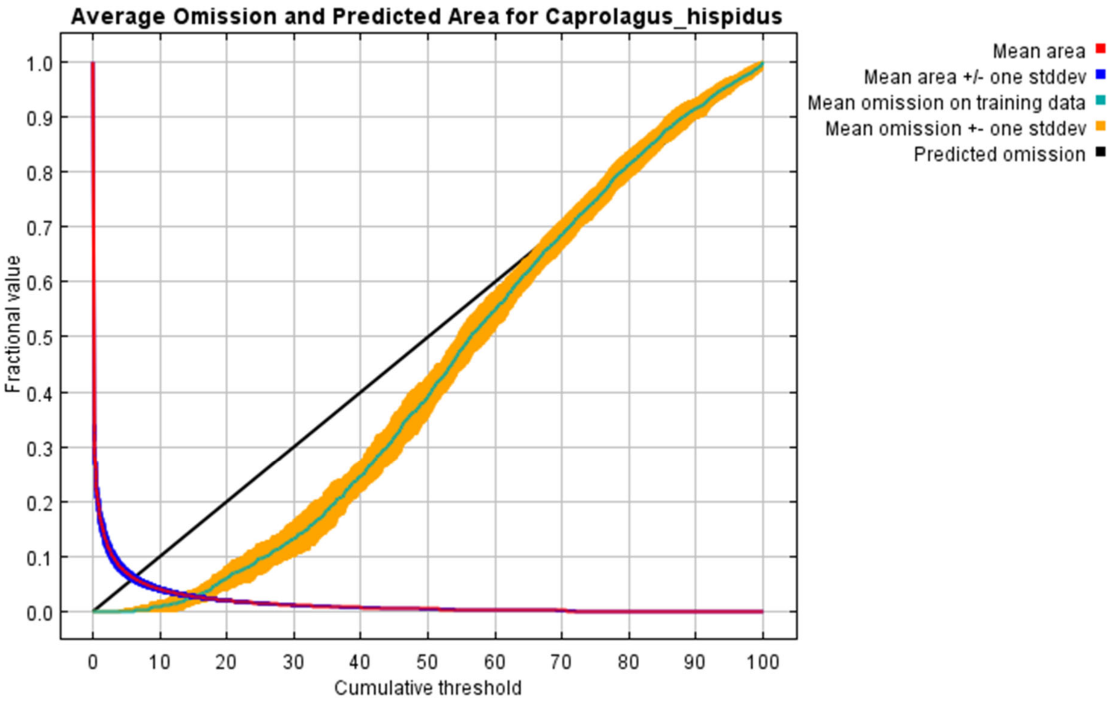

Figure S2. The training omission rate and predicted area as a function of the cumulative threshold, averaged over the 20 replicate runs.

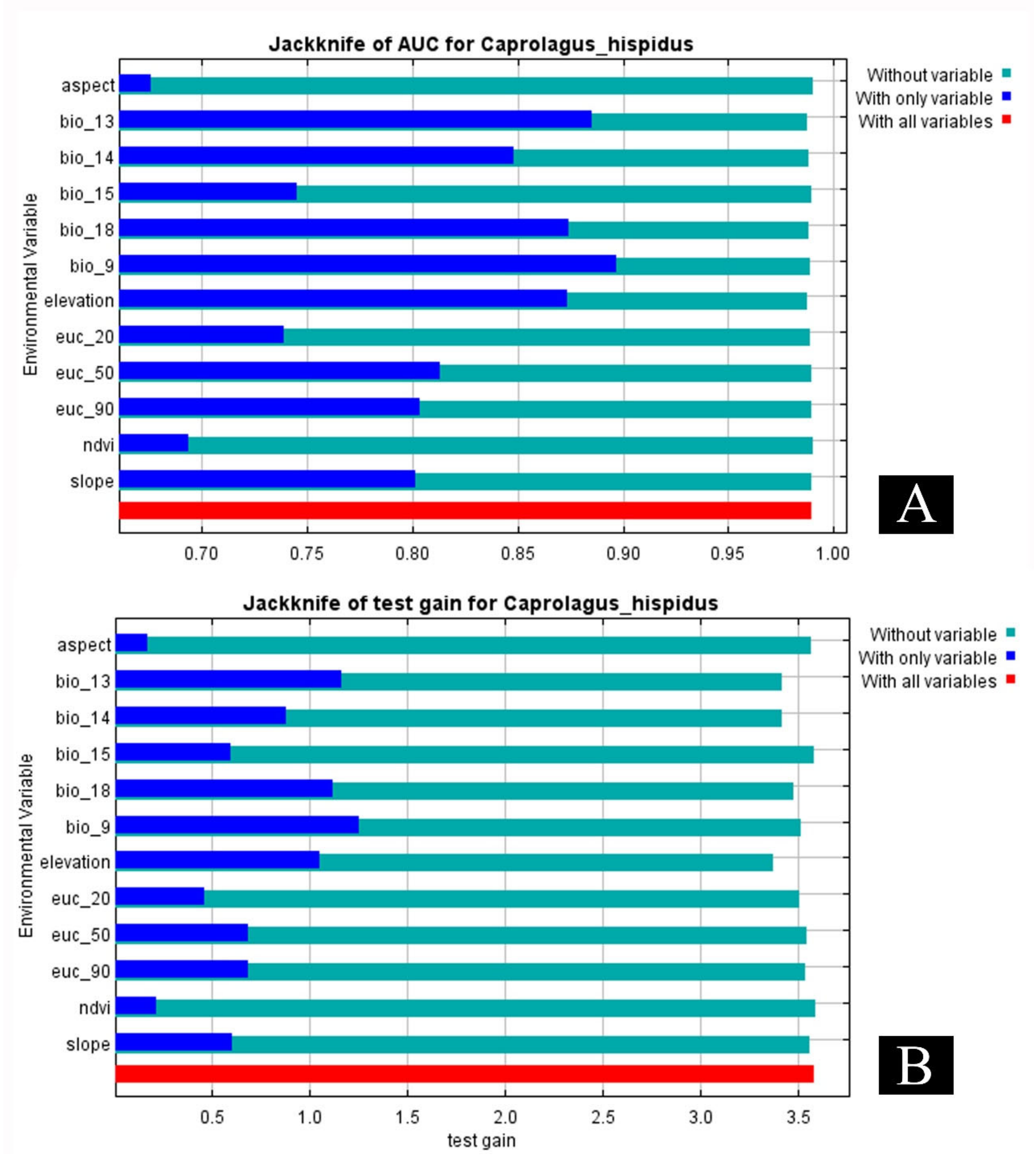

Figure S3. (A) The image shows the jackknife test, using AUC on test data. (B) The image shows the jackknife test of test gain.

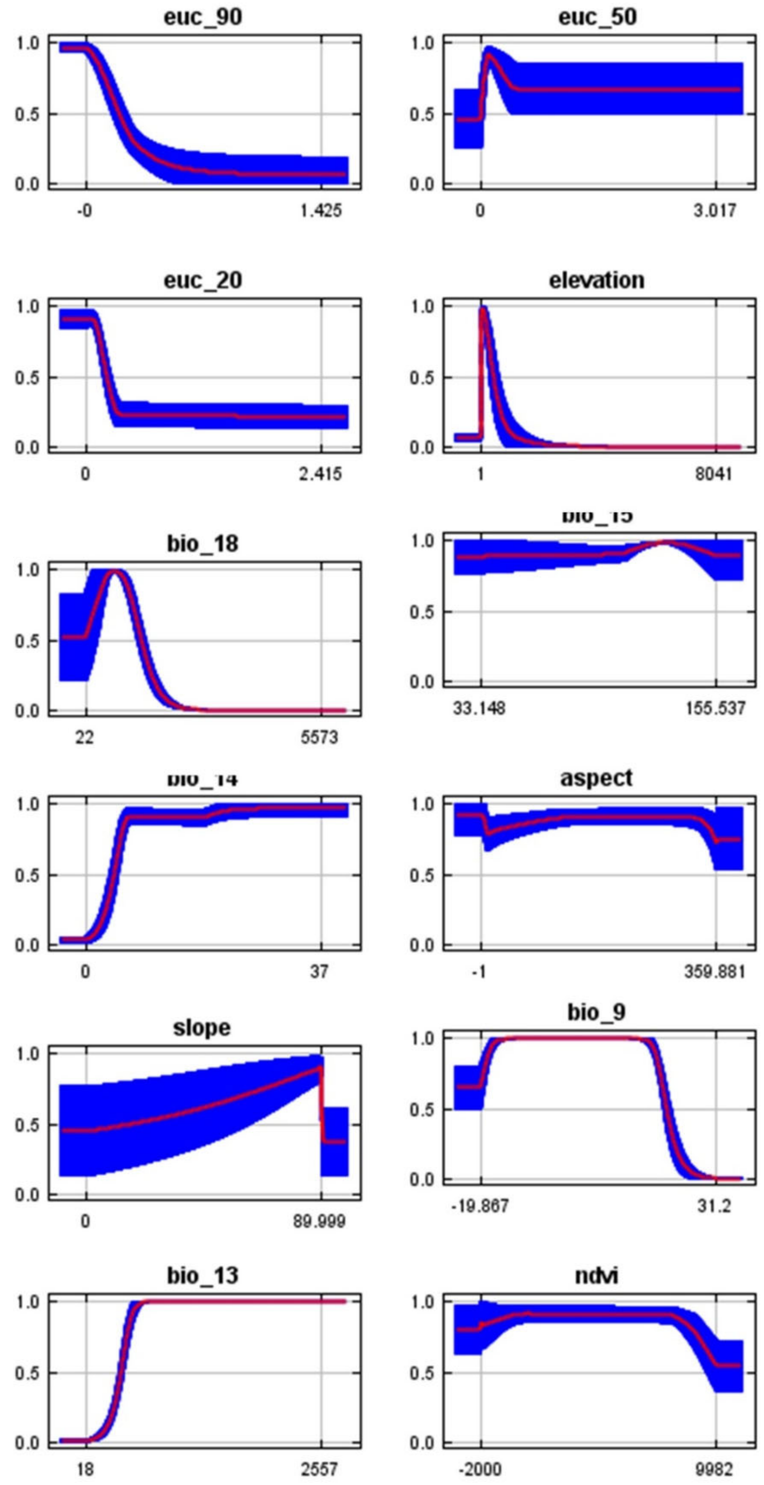

Figure S4. The curves show how each environmental variable affects the model prediction and how the predicted probability of presence changes as each environmental variable is varied, keeping all other environmental variables at their average sample value. It also shows the mean response of the 20 replicate MaxEnt runs (red) and the mean +/- one standard deviation (blue, two shades for categorical variables).

Table S1. Percentage contribution and permutation contribution with covariates details.

Variable	Variable Code	Percent contribution	Permutation importance
Precipitation of Warmest Quarter	bio_18	28.4	6.3
Slope	slope	16.4	1.7
Precipitation of Driest Month	bio_14	11.6	7.9
Elevation	elevation	9.4	45.6
Euclidean Distance to Herbaceous wetland (Lands with a permanent mixture of water and herbaceous or woody vegetation. The vegetation can be present in either salt, brackish, or fresh water)	euc_90	7.6	3
Precipitation of Wettest Month	bio_13	6.8	13.6
Precipitation Seasonality (Coefficient of Variation)	bio_15	5.9	0.6
Euclidean Distance to Urban/built up (Land covered by buildings and other man-made structures)	euc_50	5.6	1.2
Euclidean distance to Shrubs (woody perennial plants with persistent and woody stems and without any defined main stem being less than 5 m tall. The shrub foliage can be either evergreen or deciduous.)	euc_20	4.5	2.3
Mean Temperature of Driest Quarter	bio_9	1.6	17.5
Aspect	aspect	1.3	0.3
Normalized difference vegetation index	ndvi	0.9	0.1

Table S2. Estimated suitable habitat (in km^2) in different climate change scenarios.

Scenarios	Area (In sq. km.)
Present	11374
SSP 126 (2041-2060)	10620
SSP 126 (2061-2080)	9969
SSP 245 (2041-2060)	10440
SSP 245 (2061-2080)	5457
SSP 585 (2041-2060)	8242
SSP 585 (2061-2080)	4291

Table S3. Protected Areas in the distribution range of *C. hispidus,* out of which top 20 are demonstrated in Table 1. NP: National Park; WLS: Wildlife Sanctuary.

Sl. No.	Country	State/Province	NAME	Mean Suitability (Present)
1	Nepal	Mahakali Province	Shuklaphanta NP	0.837
2	India	Assam	Dibru-Saikhowa NP	0.631
3	India	Assam	Orang NP	0.572
4	India	Uttarakhand	Corbett NP	0.530
5	India	Arunachal Pradesh	D'Ering Memorial WLS	0.477
6	India	Uttar Pradesh	Dudhwa NP	0.464
7	India	Assam	Kaziranga NP	0.463
8	Nepal	Bagmati Province	Chitawan NP	0.446
9	India	Assam	Burachapori WLS	0.437
10	India	Uttarakhand	Sonanadi WLS	0.423
11	Nepal	Lumbini Province	Bardia NP	0.384
12	India	Assam	Nameri NP	0.376
13	India	Assam	Laokhowa WLS	0.329
14	India	Assam	Pani-Dihing WLS	0.318
15	India	Assam	Sonai-Rupai WLS	0.266
16	India	Assam	Manas NP	0.245
17	India	Bihar	Valmiki NP	0.216
18	India	Uttar Pradesh	Katerniaghat WLS	0.192
19	India	Uttar Pradesh	Kishanpur WLS	0.108
20	India	Assam	Borail WLS	0.103
21	India	Assam	Bherjan-Borajan-Podumoni WLS	0.086
22	India	Assam	Gibbon WLS	0.077
23	India	Uttar Pradesh	Sohelwa WLS	0.069
24	India	Arunachal Pradesh	Kane WLS	0.048
25	India	Arunachal Pradesh	Sessa Orchid WLS	0.043
26	India	Arunachal Pradesh	Pakhui WLS	0.043
27	India	Assam	Chakrashila WLS	0.043
28	India	Uttar Pradesh	Sohagibarwa WLS	0.034
29	India	Uttar Pradesh	Bakhira WLS	0.031
30	Bhutan	-	Royal Manas NP	0.030
31	India	West Bengal	Buxa NP	0.019
32	India	Uttarakhand	Binsar WLS	0.017
33	India	West Bengal	Jaldapara WLS	0.016
34	India	Assam	Nambor Doigrung WLS	0.015
35	India	Arunachal Pradesh	Namdapha NP	0.014
36	India	West Bengal	Gorumara NP	0.009
37	India	Arunachal Pradesh	Mehao WLS	0.007
38	India	West Bengal	Sunderban NP	0.007
39	India	Arunachal Pradesh	Mouling NP	0.005
40	India	Uttarakhand	Kedarnath WLS	0.005
41	India	Arunachal Pradesh	Kamlang WLS	0.005
42	India	Bihar	Gautam buddha WLS	0.003
43	India	West Bengal	Ballavpur WLS	0.003
44	India	Bihar	Barela S.A.Z.S. WLS	0.003
45	India	West Bengal	Narendrapur WLS	0.003

46	India	West Bengal	Chapramari WLS	0.002
47	India	West Bengal	Mahananda WLS	0.002
48	India	Arunachal Pradesh	Itanagar WLS	0.002
49	India	Arunachal Pradesh	Tale Valley WLS	0.001
50	India	Bihar	Nagi Dam WLS	0.001
51	India	Bihar	Bhimbandh WLS	0.001
52	India	West Bengal	Neora Valley NP	0.001
53	India	Uttarakhand	Nanda Devi NP	0.001
54	India	West Bengal	Senchal WLS	0.001
55	India	West Bengal	Lothian Island WLS	0.000
56	India	Uttarakhand	Valley of Flowers NP	0.000
57	India	Bihar	Vikramshila Ganges WLS	0.000
58	India	Arunachal Pradesh	Eagle Nest WLS	0.000
59	India	Arunachal Pradesh	Yordi-Rabe Supe	0.000
60	India	West Bengal	Jorepokhri Salamander WLS	0.000
61	India	Uttar Pradesh	Sandi WLS	0.000
62	India	Uttar Pradesh	Surha Tal WLS	0.000
63	India	Bihar	Rajgir pant WLS	0.000
64	India	West Bengal	Raiganj WLS	0.000
65	India	Uttar Pradesh	Turtle WLS	0.000
66	India	Bihar	Kanwarjheel WLS	0.000
67	India	Uttar Pradesh	National Chambal WLS	0.000
68	India	Uttar Pradesh	Parvati Aranga WLS	0.000
69	India	Arunachal Pradesh	Dibang WLS	0.000
70	India	Uttar Pradesh	Lakh Bahosi WLS	0.000
71	India	West Bengal	Singalila NP	0.000
72	India	Bihar	Kaimur WLS	0.000
73	India	Uttar Pradesh	Chandraprabha WLS	0.000
74	India	Uttar Pradesh	Samaspur WLS	0.000
75	India	Uttar Pradesh	Kaimur WLS	0.000
76	India	Uttar Pradesh	Nawabganj WLS	0.000
77	India	West Bengal	Bethuadahari WLS	0.000
78	India	Uttarakhand	Gangotri NP	0.000
79	India	Uttar Pradesh	Saman WLS	0.000
80	India	Uttar Pradesh	Ranipur WLS	0.000
81	India	Uttarakhand	Govind NP	0.000
82	India	Uttar Pradesh	Vijai Sagar WLS	0.000
